# Optimizing progeny size and number of crosses under genomic selection: insights into additive and epistatic contributions to long-term genetic gain

**DOI:** 10.1007/s00122-026-05164-2

**Published:** 2026-02-01

**Authors:** Jesimiel da Silva Viana, Júlio César DoVale, Roberto Fritsche-Neto

**Affiliations:** 1 Department of Crop Science, Federal University of Ceara, Fortaleza, Ceará Brazil; 2 North Carolina State University, Raleigh, NC USA

## Abstract

**Key message:**

**Designing genomic selection to capture the additive and epistatic effects.**

**Abstract:**

Genomic selection (GS) offers great potential to accelerate long-term genetic gain, but strategic decisions such as progeny size and number of crosses remain poorly established, particularly under contrasting resource scenarios. We conducted stochastic simulations of rice breeding programs over 50 years (10 cycles) using progeny sizes of 25, 50, 100, and 200 individuals, under both theoretical (unlimited resources) and practical (budget-constrained to 4000 F_2_ individuals) contexts, and considering three levels of epistasis (absent, moderate, high). In theoretical scenarios, larger progenies consistently achieved higher gains. After 50 years, progenies of 200 individuals reached cumulative responses to selection of 2.39 (1.96% yr^−1^) with no epistasis, 3.20 (2.60% yr^−1^) under moderate epistasis, and 3.48 (3.34% yr^−1^) under high epistasis. These schemes also maximized prediction accuracy and efficiently converted additive and epistatic variance into genetic gain. Conversely, under budget constraints, smaller progenies combined with more crosses outperformed larger ones. Progenies of 25 and 50 individuals achieved the greatest responses—up to 2.58 (2.07% yr^−1^) without epistasis, 3.36 (2.76% yr^−1^) under moderate epistasis, and 2.72 (2.45% yr^−1^) under high epistasis—while maintaining higher genetic diversity across cycles. Our results demonstrate that in resource-unlimited conditions, larger progenies (200 individuals) maximize the capture of additive and epistatic effects, whereas in budget-constrained programs, smaller progenies (25–50 individuals) coupled with more crosses provide the most efficient strategy. These findings provide practical guidelines for breeders to design GS schemes that reconcile high long-term genetic gain with operational feasibility, highlighting the decisive role of epistasis in shaping gain trajectories.

## Introduction

Genomic selection (GS) has revolutionized plant breeding since its introduction by Meuwissen et al. (2001). By using genome-wide molecular markers to predict the breeding value of individuals, GS enables earlier and more accurate selection, reducing reliance on extensive phenotyping and significantly shortening breeding cycles (Jannink et al. 2010; Xu et al. 2017). This approach has already been applied in diverse crops, including rice, maize, wheat, soybean, and sugarcane (Robertsen et al. 2019; Yadav et al. 2020; Fritsche-Neto et al. 2024a), where it has contributed to increasing the rate of genetic gain per unit of time. Despite this progress, its effective use in breeding programs requires careful definition of breeding schemes, especially with respect to the design of progeny per cross and the allocation of resources across cycles.

The definition of progeny size per cross is one of the most critical and yet unresolved aspects of GS implementation. Progeny size directly influences the probability of capturing elite recombinants, the level of within-family variance expressed, and the accuracy of selection. Smaller progenies restrict the chances of recovering favorable allele combinations, thereby limiting long-term progress (Huehn 1996). Conversely, very large progenies may increase operational costs and lead to diminishing returns in gain, since additional individuals may contribute little new information after a certain point (Ramalho et al. 2013; Atanda and Bandillo 2023). In addition, progeny size has implications for the accuracy of variance component estimation, the control of genetic drift, and the sustainability of genetic gain across cycles (da Silva et al. 2025; Porto et al. 2025). These considerations are particularly important for small- and medium-scale breeding programs, where human and financial resources are limiting, making the optimization of progeny size a decisive factor for success.

Most studies on GS have emphasized additive genetic effects, which are the primary drivers of heritable variation and are effectively modeled using linear mixed models such as GBLUP (VanRaden 2008; Robertsen et al. 2019). However, non-additive effects, especially epistasis, play a significant role in the genetic architecture of complex traits and can profoundly influence long-term responses to selection (Holland 2001; Bonk et al. 2016; Dwivedi et al. 2024). Epistasis, resulting from interactions between alleles at different loci, can either enhance or reduce phenotypic expression, and its contribution to genetic variance often depends on recombination events and population structure (Tessele et al. 2025). Theoretical and empirical studies have shown that larger progenies are required to capture favorable recombinations and release additive variance masked by epistatic interactions (Zapata-Valenzuela et al. 2013; Fritsche-Neto et al. 2024b). Thus, progeny size is not only relevant to additive effects but also critical for the expression and exploitation of epistatic variance in GS-based breeding schemes.

Another practical constraint is budget. Although genotyping costs have declined, the evaluation of thousands of individuals per cycle remains a major limitation for many public and small-scale programs (Porto et al. 2025). In practice, breeders often face trade-offs between the number of crosses and the number of individuals per progeny when a fixed total number of individuals per cycle is imposed (e.g., 4000 F_2_ plants). Increasing the number of crosses broadens genetic diversity but reduces within-family sampling depth, whereas larger progenies enhance the chances of capturing recombinants within fewer crosses but at the cost of narrowing the genetic base. Optimizing this balance is therefore a central challenge, and its consequences for additive and epistatic variance remain poorly quantified.

Stochastic simulations have become a powerful tool to evaluate breeding strategies and predict long-term outcomes under different assumptions. They allow the systematic exploration of breeding design choices that would be impractical to test empirically, such as progeny size, number of crosses, training set design, and marker density (DoVale et al. 2022; Sabadin et al. 2022; Fritsche-Neto et al. 2024a; Vieira et al. 2025; Li et al. 2025). Simulation studies have clarified key aspects of GS implementation, but to date few have explicitly investigated how progeny size interacts with levels of epistasis and resource availability to shape long-term genetic gain and sustainability. This represents a critical gap, since progeny size decisions simultaneously affect additive and non-additive variance, genetic drift, and the efficiency of GS predictions across multiple cycles.

Accordingly, the objective of this study was to use stochastic simulations to assess the impact of progeny size on long-term GS efficiency. Specifically, we aimed to (i) determine the optimal progeny size under theoretical scenarios with varying levels of epistasis (absent, moderate, and high), and (ii) identify the optimal allocation of progeny size and number of crosses under budget-constrained conditions, with a fixed total of 4000 F_2_ individuals per cycle. By quantifying genetic gain, variance components, and predictive accuracy across 50 years of breeding (10 cycles), this study provides evidence-based recommendations for breeders to design GS schemes that balance additive and epistatic contributions while accounting for resource limitations.

## Methods

### Structure of the simulated breeding program

The present study was conducted based on the structure of a rice breeding program from the Louisiana State University Ag Center (da Silva et al. 2025), which was used as a model for long-term simulations. A period of 50 years was simulated, corresponding to 10 selection cycles of five years each, considering different progeny sizes and distinct levels of epistasis, in two contexts: a theoretical scenario, without budget limitations, and a practical scenario, in which the total number of F_2_ individuals was restricted to 4000 per cycle.

### Founder population and genetic parameters

The founder population consisted of 1000 diploid pure-line individuals, each with 12 pairs of chromosomes, simulated using the Markovian Coalescent Simulator–MaCS (Chen et al. 2009), considering a “GENERIC” species. The genome was segmented into 1644 blocks according to chromosome length in centiMorgans (cM). The simulated quantitative trait was controlled by 360 QTNs (30 per chromosome). In addition, 540 SNPs were uniformly distributed, ensuring that SNPs and QTNs did not overlap in position.

The genetic effects at each QTN were assigned as follows: (i) additive–sampled from a Gaussian distribution (shape and scale parameters = 1); (ii) epistatic (additive × additive)–three levels: 0 (absent), 0.5 (moderate), and 1.0 (high), representing the proportion of epistatic variance (Vaa) relative to additive variance (Va).

The genetic value of each individual i was calculated as the sum of additive and epistatic effects, according to the expression:

$${G}_{i}=\sum_{k=1}^{360}{a}_{k}{x}_{ik}+ \sum_{\left(k,l\right)}{a}_{kl}{x}_{ik}{x}_{il},$$

where ${a}_{k}$ corresponds to the additive effect of QTN $k$, ${a}_{kl}$ to the epistatic effect between QTNs $k$ and $l$, and ${x}_{ik}$ to the scaled genotypic dosage of individual $i$. The phenotype was obtained as the sum of an intercept $\mu$, the total genetic value, and the random error, according to:

$${Y}_{i}=\mu +{G}_{i}+{\varepsilon }_{i},{\varepsilon }_{i}\sim \mathcal{N}(0,{\sigma }_{e}^{2})$$

where ${\sigma }_{e}^{2}$ was adjusted to ensure a broad-sense heritability of approximately 0.63 in the initial cycle.

### *Burn-in* phase and base population

**Fig. 1:**
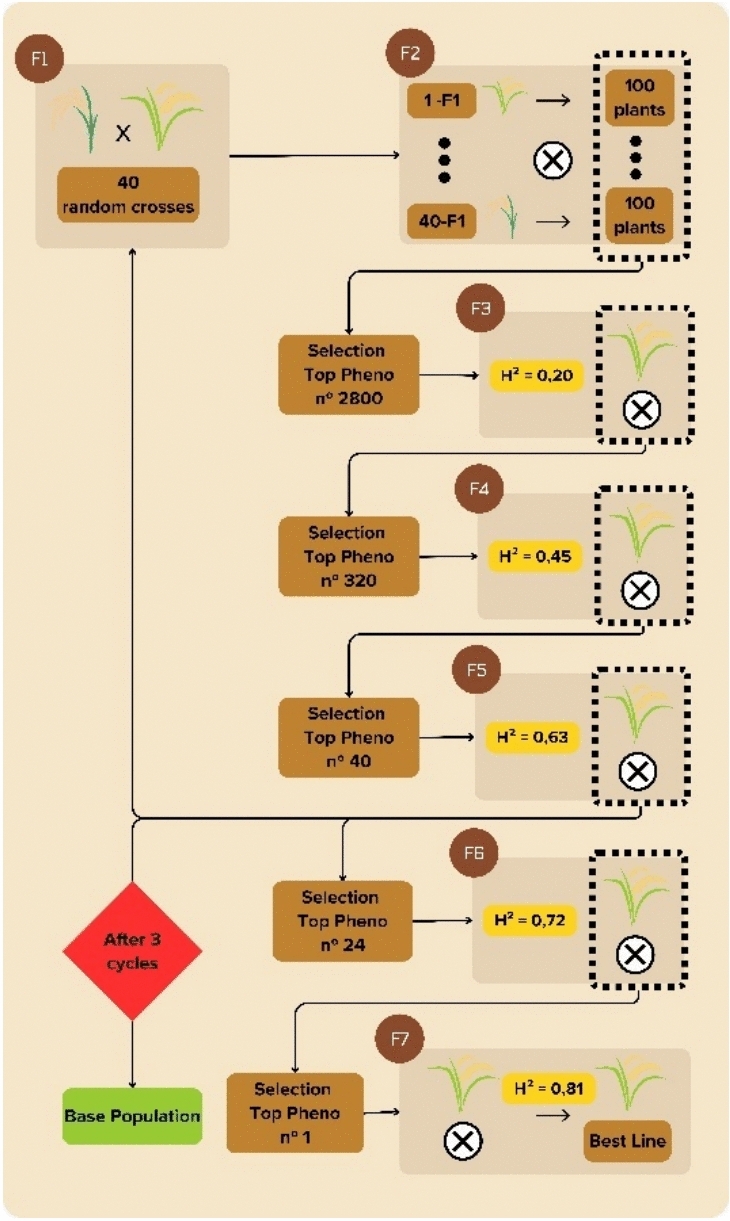
Traditional scheme of a breeding program used in the *burn-in* phase

In the initial phase, the traditional pedigree method was used (Breseghello and Coelho 2013), simulating 160 random crosses advanced to the F_2_ generation, with 100 individuals per progeny (Fig. 1). From F_2_ to F_6_, variable selection intensities were applied, selecting new parents in F5 and the best line in F6. Each cycle lasted 5 years, totaling 15 years of *burn-in* for the establishment of the base population at each evaluated level of epistasis.

### Simulations with different progeny sizes

**Fig. 2:**
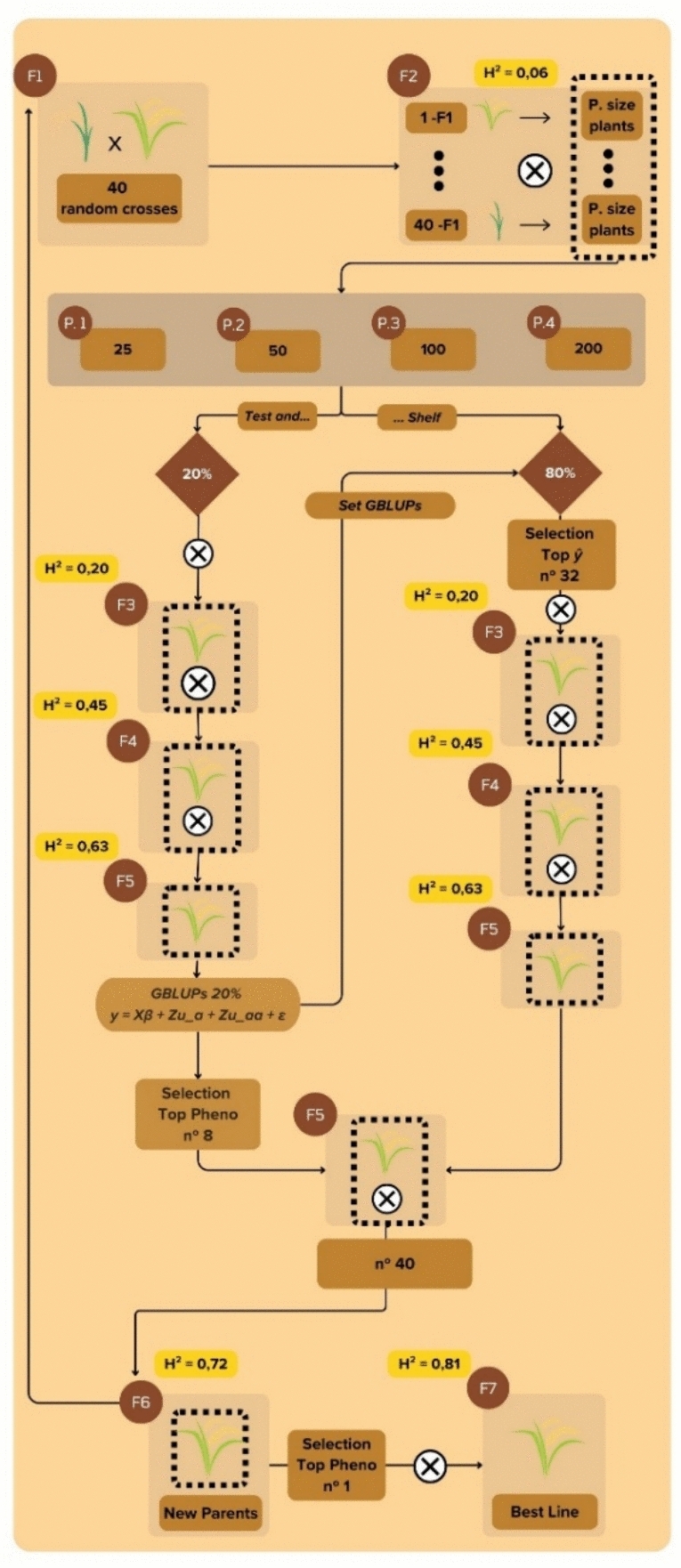
Breeding scheme used to simulate 50 years of a program under genomic selection

Once the base populations for each evaluated level of epistasis were obtained, 50 years of a breeding program with genomic selection were simulated (Fig. 2). The breeding scheme was based on the pedigree method with adjustments to GS. Initially, 40 random crosses among 40 parents were performed. Each cross was advanced to F_2_ under different progeny sizes: 25, 50, 100, or 200. In F_2_, GS was applied using the “test and shelf” method. At this stage, the population was divided into two groups. The first, defined as *test*, corresponded to a random selection of 20% of the population. The second group, referred to as *shelf*, consisted of the remaining 80%.

The individuals from the test group were first advanced through selfing to F5, where, based on genotypic and phenotypic information, the Genomic Best Linear Unbiased Predictors (GBLUPs) of their additive and non-additive effects were obtained. Once the GBLUPs of the 20% were predicted, the remaining 80% of the population had their total genetic values estimated in F_2_. The applied selection intensity corresponded to 80% of the total number of parents to be obtained. As in the test group, the individuals selected in shelf were also advanced to F_5_ through successive selfings.

Finally, in F_5_, the eight best individuals from the test group were chosen based on phenotypic values. These individuals were then combined into a single group together with those selected in *shelf*, and all were again selfed to obtain new parents in F_6_. From F_6_, the best line was selected and carried forward to the subsequent selection cycle. It is worth noting that the previously described steps corresponded to the theoretical context. However, we also analyzed a practical context of budgetary limitations in the breeding program, in which the maximum number of individuals in F2 was set at 4000. Thus, to determine the best progeny size among 25, 50, 100, and 200, it was necessary to adjust the number of initial crosses to 160, 80, 40, and 20, respectively.

### Genomic prediction

Genomic prediction was performed using the GBLUP model, which was fitted with a training set composed of 20% of the F_2_ individuals. The choice of this method was due to its lower computational cost, especially for models involving additive and non-additive effects such as epistasis.

The SNP genotypic coding followed the convention − 1, 0, 1 for genotypes A_2_A_2_, A_1_A_2_, and A_1_A_1_, respectively. The genotype matrix $G$ was centered, resulting in $M$, according to:

$$M=G-{1}_{n}{\overline{x} }^{\top }, {\overline{x} }_{k}=\frac{1}{n}\sum {x}_{ik},$$

where $M$ is the centered marker matrix obtained by subtracting the mean of each marker in matrix $G,$ of dimension *n* x *m*, with *n* individuals and *m* markers.

The additive genomic relationship matrix ($${G}_{a})$$ was calculated according to VanRaden (2008):

$${G}_{a}= \frac{M{M}^{\top }}{\sum_{k=1}^{m}{d}_{k}}, { d}_{k}={\left({M}_{.k}\right)}^{\top }\left({M}_{.k}\right),$$

where ${d}_{k}$ is the value on the diagonal of the product $M{M}^{\top }$.

Once the ${G}_{a}$ matrix was obtained, the epistatic genomic relationship matrix $({G}_{aa})$ was generated by the Hadamard product, multiplying each cell of the additive genomic relationship matrix by itself, as follows:

$$G_{aa} = G_{a} ^\circ G_{a}$$

Subsequently, the normalized epistatic genomic relationship matrix ${({\boldsymbol{G}}}_{{\boldsymbol{a}}{\boldsymbol{a}}\boldsymbol{^{\prime}}}$**)** was obtained to ensure that the mean of the diagonal was equal to 1, using the expression:

$$G_{aa^{\prime}} = \frac{{G_{aa} }}{{\left( {{\raise0.7ex\hbox{${tr \left( {G_{aa} } \right)}$} \!\mathord{\left/ {\vphantom {{tr \left( {G_{aa} } \right)} {n }}}\right.\kern-0pt} \!\lower0.7ex\hbox{${n }$}}} \right)}}$$

where $tr ({G}_{aa})$ is the trace of the ${G}_{aa{\prime}}$ matrix and $n$ is the number of individuals in the matrix.

With the matrices, the GBLUP-based models had their variance and covariance components estimated by the restricted maximum likelihood (REML) method using the Newton–Raphson (NR) algorithm, implemented through the sommer-R package (version 4.3.7) (Covarrubias-Pazaran 2016). For this, the following was used:

$$y=X\beta +{Z}_{a}a {+ Z}_{aa}aa+ \varepsilon$$

where $y$ is the vector of phenotypic values; $\beta$ the vector of fixed genotype effects; ***a*** the vector of random additive effects, with $a \sim N\left(0,{G}_{a}{\sigma }_{a}^{2}\right)$; *aa* the vector of random additive × additive epistatic effects, with $aa \sim N\left(0,{G}_{aa}{\sigma }_{aa}^{2}\right)$*; ε* is the random residual effect; *X* the incidence matrix of fixed effects; *Z*_*a*_ and *Z*_*aa*_ the incidence matrices for random additive and additive × additive epistatic effects, respectively.

After fitting the models, the GBLUP values of additive and epistatic genetic effects were extracted. These two components were summed to form the total predicted genetic value of the phenotyped individuals. In addition, the intercept of the model, corresponding to the mean fixed effect, was extracted, which was necessary to adjust the predicted values to the phenotype scale. Subsequently, the genotypic information of the remaining 80% of the population (shelf) was obtained, and two genomic relationship matrices were constructed: (i) the matrix between test (20%) and shelf (80%) individuals, and (ii) the matrix among test (20%) individuals. From these matrices, the total genetic values (additive + epistatic) estimated for the test group were projected onto the shelf individuals. This projection used the kinship relationship between the two groups, following the principle of prediction by genomic similarity. Finally, the phenotypic values of the 80% individuals were estimated by summing the projected genetic value with the estimated model intercept, according to the equation:

$$\widehat{y}=mu {+ GBLUP}_{a+aa}$$

where $\widehat{y}$ is the estimated phenotypic value; $mu$ is the estimated intercept of the model; ${GBLUP}_{a+aa}$ is the projected genetic value.

### Comparison of different scenarios

All scenarios in each evaluated context were simulated over 10 breeding cycles, with 100 independent replicates, using the AlphaSimR package (Gaynor et al. 2021). Since all treatments essentially employed the same simulation framework, there were no differences in the duration of the selection cycles among them, allowing for a direct comparison of the results obtained.

In each cycle, the mean true genetic value of the populations, the true genetic value of the best line, the additive variance, and the additive × additive epistatic variance were evaluated. However, since the scenarios with absence of epistasis, moderate epistasis, and high epistasis originated from different base populations, the absolute values of these variables were not directly comparable. To correct this limitation, all metrics were relativized with respect to the initial cycle of each scenario, being expressed as proportions of increase or decrease over the cycles, taking the value of cycle 0 as reference.

Prediction accuracy and response to selection were also considered fundamental metrics for comparison. Accuracy was estimated using the Pearson correlation between true genetic values (${G}_{i}$) and predicted genetic values (${\widehat{G}}_{i}$), according to the expression:

$$\rho =cor({G}_{i, }{\widehat{G}}_{i})$$

The response to selection (${R}_{t}$) was defined as the difference between the mean genetic value of the parents in cycle $t$ and the mean genetic value of the population in the initial cycle:

$${R}_{t}={\overline{G} }_{t}^{Parents}-{\overline{G} }_{0}^{Population}$$

In addition, the annualized rate of relative response was calculated, expressed as a percentage, which considers the accumulated gain relative to the initial population adjusted for the 50-year simulation horizon:

$${R}_{t}^{(\%/year)}=\frac{100}{t}\times \frac{{\overline{G} }_{t}- {\overline{G} }_{0}}{\left|{\overline{G} }_{0}\right|}$$

These combined metrics allowed the evaluation not only of the mean gains obtained over the cycles, but also of the maintenance of genetic variability, the accuracy of genomic prediction, and the relative efficiency of different progeny sizes under contrasting contexts of resource availability and epistasis intensity.

## Results

### Progeny size in the theoretical context

**Fig. 3:**
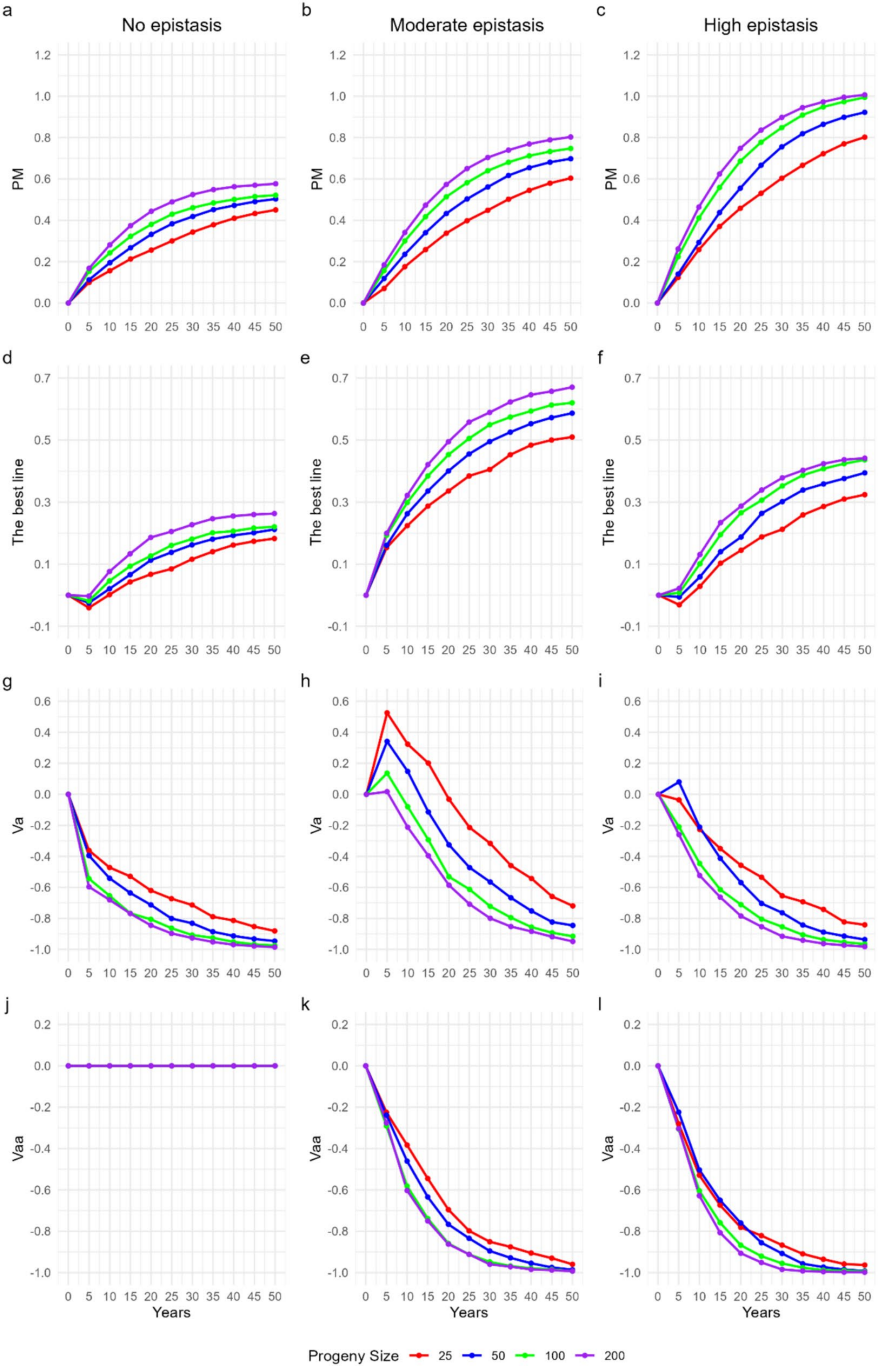
Population mean performance (**a–c**), best line (**d–f**), additive variance (**g–i**), and additive × additive variance (**j–l**) over 50 years of a breeding program under genomic selection cycles

In the theoretical scenario, population gains increased consistently as progeny size was expanded over the 50 simulated years (Fig. 3a–c). After 10 breeding cycles, progenies with 200 individuals per cross in F_2_ reached increments of approximately 58%, 80%, and 100% in the contexts with absence of epistasis, moderate epistasis, and high epistasis, respectively. These results show that under stronger influence of epistatic effects, genetic gains become more pronounced.

The performance of the best lines was also favored by the increase in progeny size (Fig. 3d–f). In populations with larger progeny size, the gains were 26% in the scenario with absence of epistasis, 70% under moderate epistasis, and 44% under high epistasis, confirming that the greatest advances occurred in the scenario with moderate epistasis.

Additive variance (Va) showed distinct behaviors across contexts (Fig. 3g–i). In populations with only additive effects, Va decreased rapidly from the first cycle, especially in larger progenies. Under moderate epistasis, the same trend was observed, but only from the fifth year onward, since in the first cycle there was an increase in Va inversely proportional to progeny size (52%, 34%, 14%, and 2% for progenies of 25, 50, 100, and 200 individuals, respectively). In the scenario with high epistasis, Va remained relatively stable in the first cycles: progenies with 25 and 50 individuals showed small initial variations (+ 8% and –4%, respectively), whereas progenies of 100 and 200 individuals exhibited abrupt reductions. Overall, the largest reductions in Va occurred in the absence of epistasis, followed by high epistasis and, lastly, moderate epistasis.

Additive × additive epistatic variance (Vaa) also showed significant reductions over the cycles in all scenarios where it was present (Fig. 3j–l). Under moderate epistasis, the consumption of Vaa was practically identical between progenies of 100 and 200 individuals, whereas under high epistasis the same pattern occurred during the first 20 years and reappeared after 40 years of selection. In the first two decades under high epistasis, progenies of 50 individuals showed a less drastic reduction of Vaa compared with the other sizes. Only from the 25th year onward was it possible to clearly observe the trend of greater consumption of Vaa associated with larger progenies.

### Progeny size under budget constraint

**Fig. 4:**
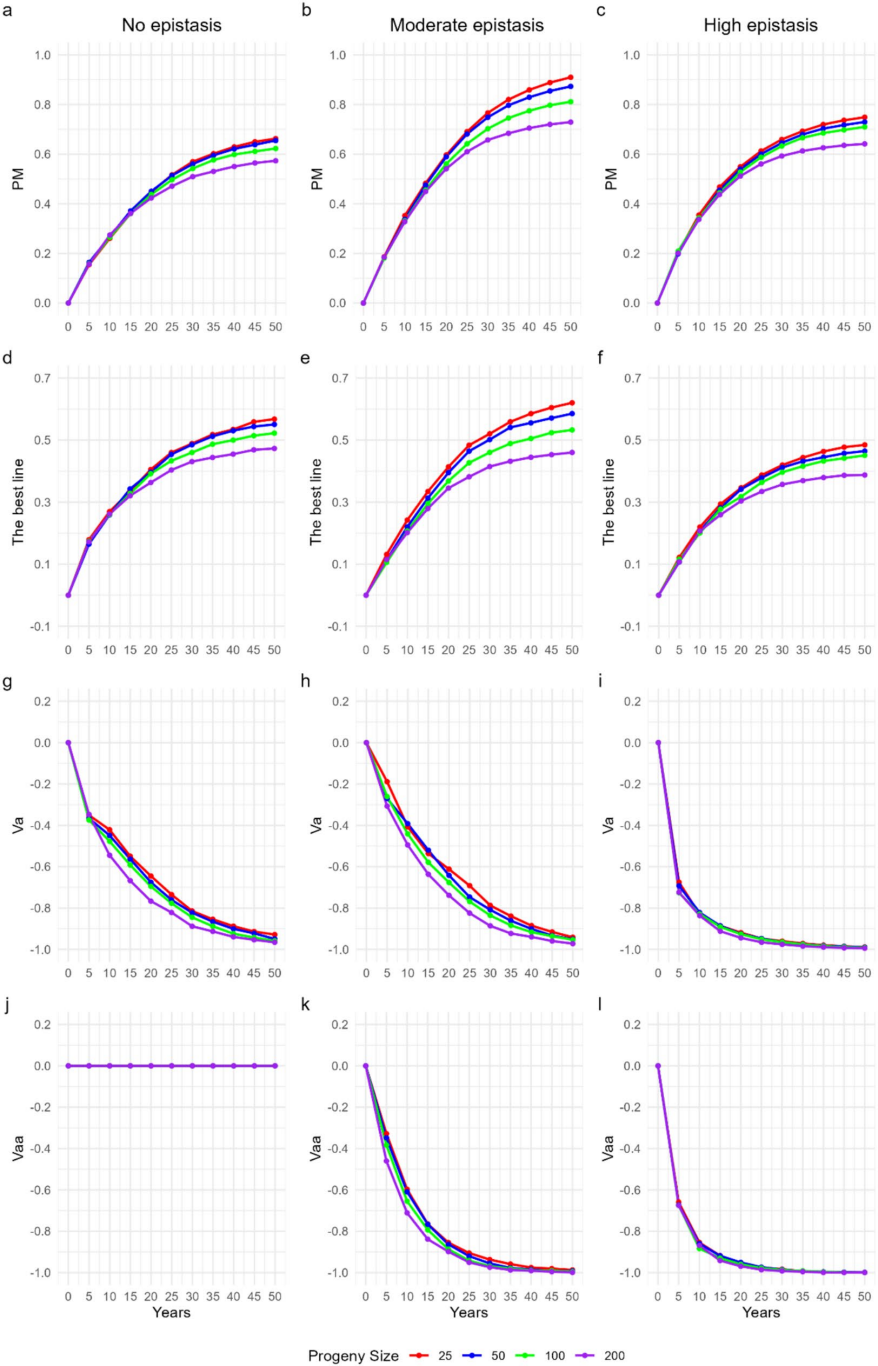
Population mean performance (**a–c**), best line (**d–f**), additive variance (**g–i**), and additive × additive variance (**j–l**) over 50 years of a breeding program under genomic selection cycles and budget limitation

In the resource-constrained scenario, the pattern observed was the opposite of the theoretical one: smaller progeny sizes resulted in greater population gains (Fig. 4a–c). After 50 years, progenies of 25 individuals provided increments of 66%, 91%, and 75% in the scenarios with absence of epistasis, moderate epistasis, and high epistasis, respectively. These effects were more pronounced in the medium and long term, with the magnitude of gains being greater under moderate epistasis, followed by high epistasis and by the absence of epistasis.

The best lines were also consistently associated with smaller progenies (Fig. 4d–f). At the end of the simulated period, progenies of 25 individuals achieved gains of 57%, 62%, and 49% in the three genetic scenarios, respectively. The temporal behavior, however, showed singularities. In the absence of epistasis scenario, from the 15th year onward, lines derived from progenies of 100 and 200 individuals started to perform worse than those from 25 and 50, with a slight advantage of the 25-individual progenies from the 20th year onward. In the scenario with moderate epistasis, the superiority of the 25-individual progenies was evident as early as the second cycle, followed by a decreasing hierarchy of 50, 100, and 200 individuals. Under high epistasis, the difference among progeny sizes became evident from the tenth year onward, consolidating the superiority of smaller progenies over time.

Va was also strongly affected by the different progeny sizes (Fig. 4g–i). In the absence of epistasis scenario, small reductions were observed as early as the first cycle, but clearer differences among sizes emerged from the fifth year onward, when larger progenies began to deplete Va more rapidly. A similar trend was observed under moderate epistasis, with lower consumption of Va in the 25-individual progenies during most of the period, whereas the 200-individual progenies showed greater losses. In the scenario with high epistasis, progenies of 25, 50, and 100 individuals displayed similar patterns of Va consumption, but 200-individual progenies led to an almost complete reduction of additive variance by the 35th year of selection.

Vaa showed distinct behaviors across levels of epistasis. Under moderate epistasis, smaller progenies (25 and 50 individuals) maintained Vaa for a longer time, whereas larger progenies accelerated its consumption, leading to complete depletion around 35 years (Fig. 4k). Under high epistasis, Vaa was completely consumed in about 30 years, regardless of progeny size (Fig. 4l).

### Accuracy of predictive models

**Fig. 5:**
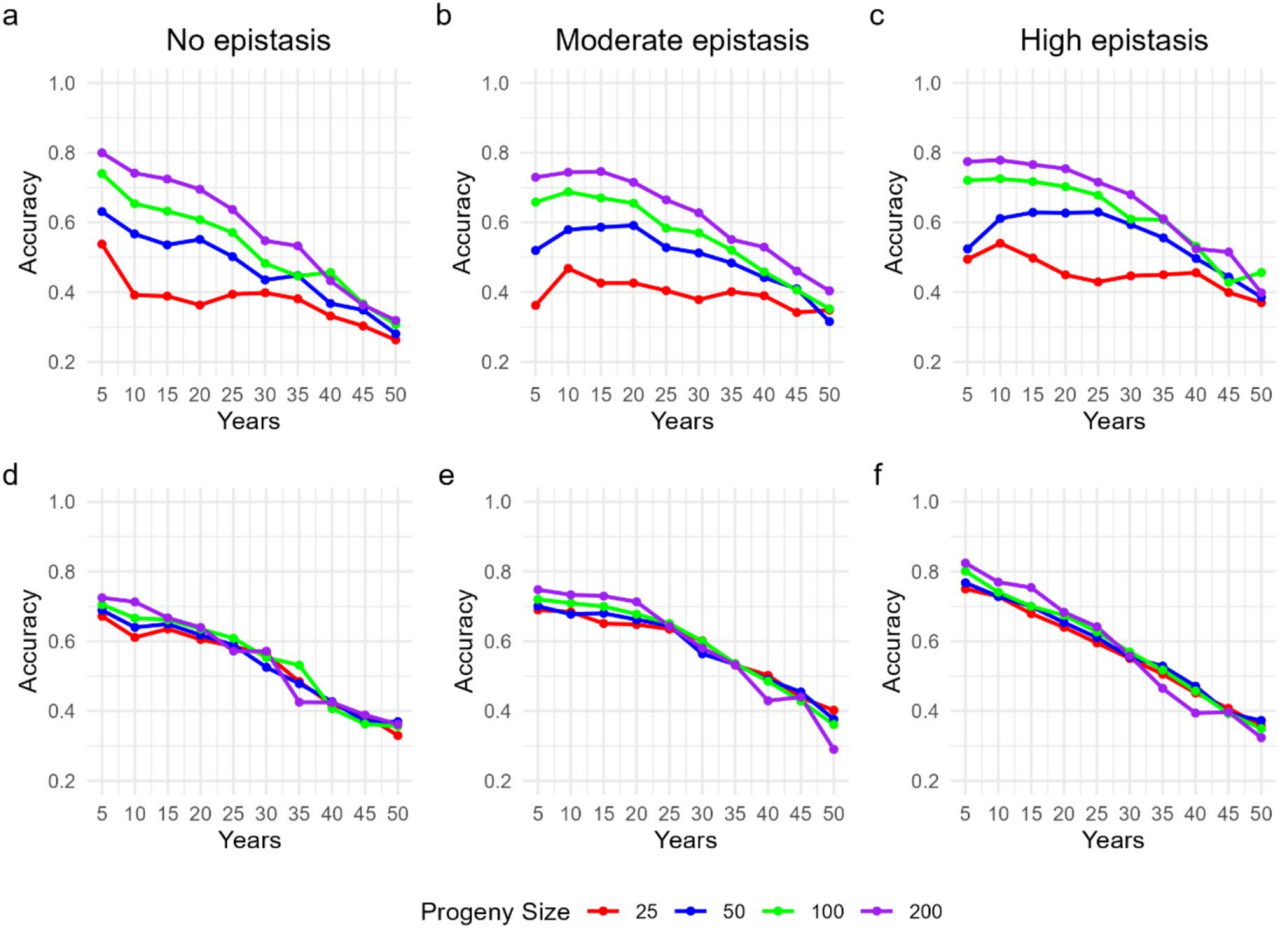
Accuracy of GBLUP-based genomic selection models over 50 years of a breeding program without and under budget limitation

In the theoretical context, the accuracy of the GBLUP models was positively influenced by the increase in progeny size (Fig. 5). In the scenario with absence of epistasis, progenies of 50, 100, and 200 individuals maintained higher accuracy until about 35 years, when they began to decline. Progenies of 25 individuals showed sharper reductions starting as early as the tenth year. Under moderate epistasis, an initial increase in accuracy was observed in the second cycle, more pronounced in smaller progenies, followed by a faster decline in intermediate sizes (50 and 100 individuals). Under high epistasis, accuracy also increased during the first 10 years, especially in progenies of 25 and 50 individuals, but declined after the 25th year. Overall, scenarios with high epistasis showed less pronounced reductions in accuracy over time compared with the others.

In the budget-constrained scenario, larger progenies maintained higher accuracy in the initial cycles (Fig. 5d-e). However, from the medium term onward, the differences among sizes became less predictable, and in all contexts, accuracy showed a linear decline trend as the cycles progressed.

### Response to selection

**Fig. 6:**
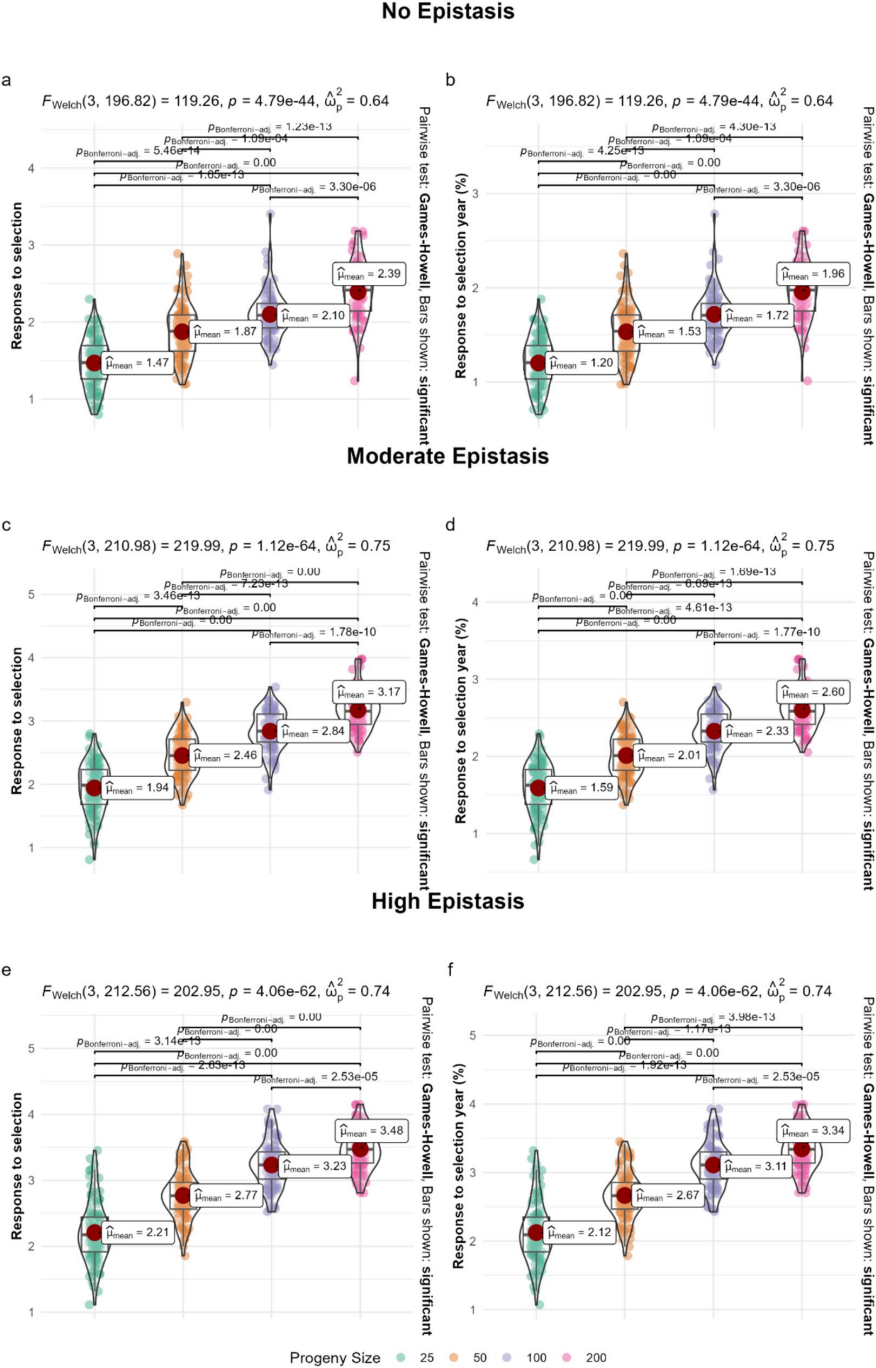
Response to selection in a breeding program under genomic selection after 50 years. Rows show epistasis level (no, moderate, high) and columns show the response metric: left (**a**, **c**, **e**) total response after 50 years; right (**b**, **d**, **f**) response per year (%). Distributions across simulation replicates are shown for progeny sizes 25, 50, 100 and 200; red points indicate means. Welch’s ANOVA and Games–Howell pairwise tests (Bonferroni-adjusted) are reported

Regardless of the genetic architecture, the increase in the number of individuals per cross resulted in greater responses to selection in the theoretical context (Fig. 6). In the scenario with absence of epistasis, mean gains ranged from 1.47 (25 individuals) to 2.39 (200 individuals) (Fig. 6a). Under moderate epistasis, values ranged from 1.94 to 3.20 (Fig. 6c), and under high epistasis, from 2.21 to 3.48 (Fig. 6e). In all cases, progeny sizes differed significantly from each other. The magnitude of the contribution of progeny size to the total variation in gains was approximately 64% in the scenario with absence of epistasis (${\upomega }_{p}^{2}=0.64)$, 75% in the scenario with moderate epistasis (${\upomega }_{p}^{2}=0.75)$ and and 74% under high epistasis (${\upomega }_{p}^{2}= 0.74)$.

The annual percentage gains followed the same trend, increasing proportionally with progeny size (Fig. 6b, d, f). In the scenario with the absence of epistasis, progenies of 200 individuals resulted in an average annual gain of 1.96% (Fig. 6b). Under moderate epistasis, this value reached 2.60% (Fig. 6d). In contrast, under high epistasis, it reached 3.34% (Fig. 6f). In all scenarios, the differences among progeny sizes were statistically significant. The proportion of variation explained by the “size” factor was consistent with that observed for the absolute values of response to selection.

**Fig. 7:**
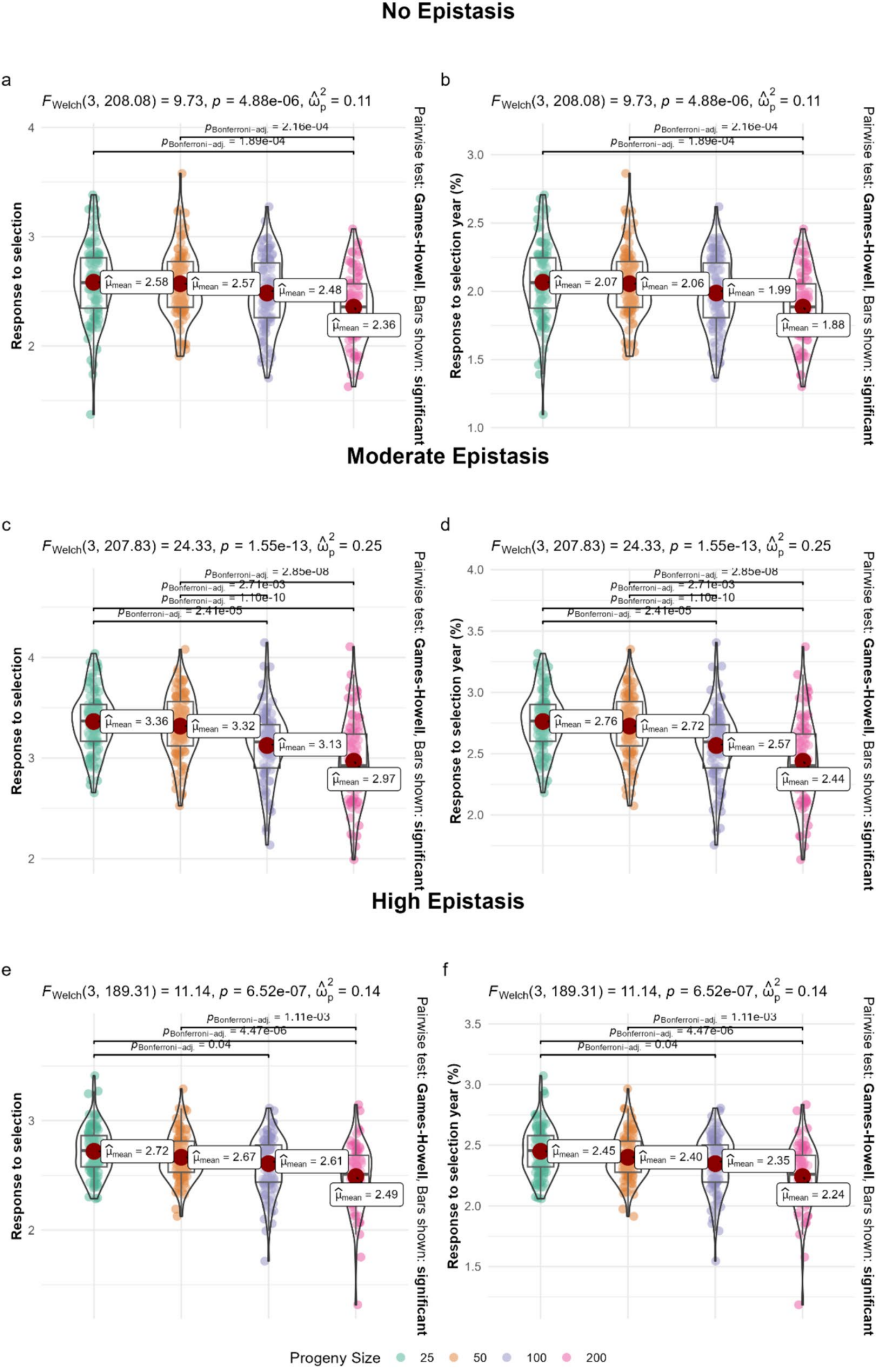
Response to selection in a breeding program under genomic selection and budget constraint after 50 years. Rows show epistasis level (no, moderate, high) and columns show the response metric: left (**a**, **c**, **e**) total response after 50 years; right (**b**, **d**, **f**) response per year (%). Distributions across simulation replicates are shown for progeny sizes 25, 50, 100 and 200; red points indicate means. Welch’s ANOVA and Games–Howell pairwise tests (Bonferroni-adjusted) are reported

In the budget-constrained scenario, the behavior was different from that observed in the theoretical context. In the scenario with absence of epistasis, mean gains ranged from 2.36 (200-individual progenies) to 2.58 (25-individual progenies), with only 11% of the total variation attributed to progeny size (${\upomega }_{p}^{2} = 0.11)$; (Fig. 7a). Under moderate epistasis, the values ranged from 2.97 to 3.36, with 25% of the variation explained by progeny size (${\upomega }_{p}^{2}= 0.25)$; (Fig. 7c). Under high epistasis, the range was 2.49–2.72, with 14% of the variation explained (${\upomega }_{p}^{2} = 0.14)$; (Fig. 7e). In all practical scenarios, the differences among progeny sizes were statistically significant, with the arrangements of 25 and 50 individuals consistently showing the highest responses to selection.

The annual percentage gains under the budget constraint confirmed this trend (Fig. 7b, d, f). In the scenario with absence of epistasis, the highest values were 2.07% and 2.06% per year for progenies of 25 and 50 individuals, respectively (Fig. 7b). Under moderate epistasis, the percentages reached 2.76% and 2.72% (Fig. 7d). Under high epistasis, the annual gains were 2.45% and 2.40% for progenies of 25 and 50 individuals, respectively (Fig. 7f). Thus, both in absolute values and percentages, smaller progenies showed superior performance, confirming the strategic advantage of reducing progeny size when resources are limited.

## Discussion

### Theoretical progeny sizes and long-term gains

In theoretical scenarios without resource constraints, progeny size proved decisive for maximizing long-term genetic gain. Progenies of 200 individuals achieved cumulative responses nearly 50% higher than progenies of 25 individuals after 50 years, across all levels of epistasis (Fig. 3a–f). This advantage stems from the increased probability of capturing superior recombinants within larger families, which elevates the likelihood of retaining rare but favorable allelic combinations. The relationship between progeny size and genetic gain has been noted since early quantitative genetic studies, with Huehn (1996) and Ramalho et al. (2013) demonstrating that larger progenies increase the potential to identify superior genotypes, even under conventional pedigree breeding.

Our findings also reinforce the importance of progeny size under epistatic architectures. Gains from larger progenies were most pronounced when epistasis was moderate to high, with responses exceeding 3.20 after 10 cycles (Fig. 6). This suggests that progeny size facilitates the release of hidden additive variance through recombination, enabling the expression of favorable epistatic interactions. This mechanism is consistent with the theoretical models of Cheverud and Routman (1999) and the empirical evidence summarized by Bonk et al. (2016), both of which emphasize that epistasis can contribute to long-term gain if sufficient recombination events are captured.

These results imply that in resource-unlimited contexts, breeders should prioritize larger progenies when designing genomic selection (GS) pipelines. By expanding sampling depth, larger progenies maximize selection opportunities within families, thereby enhancing the conversion of genetic variance into realized gain. This principle has also been highlighted in recent simulation studies with maize and wheat (Atanda and Bandillo 2023; Fritsche-Neto et al. 2024a), supporting its generalization across self-pollinated and cross-pollinated crops.

### Variance components and recombination dynamics

The depletion of additive variance (Va) observed across cycles is an expected outcome of recurrent selection, yet progeny size modulated its rate of decline. Smaller progenies maintained Va for longer than larger ones (Fig. 3g–i). Although Va depletion was evident in progenies of 200 individuals, the faster loss of Va in larger progenies likely reflects more effective selection. In line with the classical principle that the probability of capturing favorable allele combinations is proportional to the number of individuals per family (Falconer and Mackay 1996; Bernardo 2020), larger progenies tended to project greater gains across cycles, albeit with a higher consumption of Va. Thus, progeny size is not only a determinant of immediate genetic gain but also of the sustainability of variance components across cycles.

The interaction between additive and epistatic variance further illustrates this dynamic (Fig. 3g–l). Under moderate and high epistasis, epistatic variance (Vaa) contributed substantially in the first cycles but decreased over time, coinciding with partial conversion into Va through recombination. This process, widely discussed in quantitative genetics (Holland 2001; Zapata-Valenzuela et al. 2013; Tessele et al. 2025), is facilitated when progeny sizes are large enough to generate sufficient recombination events. In our simulations, progenies of 200 individuals exhibited the clearest evidence of this conversion, sustaining greater cumulative responses.

These results highlight progeny size as a critical driver of recombination efficiency. In our context, larger progenies tended to deplete Va more rapidly, likely by accelerating allele fixation, whereas smaller progenies retained Va for longer, maintaining variance availability across cycles. Notably, Va was effectively associated with greater genetic gains, consistent with the stronger responses projected for larger progenies. This pattern also relates to empirical maize and rice programs, where maintaining larger populations has been linked to reduced drift and prolonged variance availability (Bernardo 2020; Xu et al. 2017). Therefore, progeny size directly influences both the pace and durability of genetic gain by shaping the trajectory of variance components.

### Prediction accuracy and training set design

Prediction accuracy declined across all scenarios, a consequence of decreasing linkage disequilibrium (LD) between markers and QTLs as recombination accumulated. Nevertheless, larger progenies consistently sustained higher accuracies than smaller ones (Fig. 5). After 10 cycles under moderate epistasis, accuracy was 0.40 for progenies of 200 individuals compared with only 0.35 for progenies of 25 (Fig. 5b). These differences reflect the stronger training sets (TS) generated by larger progenies, which improve the representativeness of allelic variation in genomic prediction models.

Training set updating was crucial to these outcomes. We applied the “grandparents–parents–offspring” strategy, which has been reported as one of the most effective for maintaining GS accuracy over multiple cycles (Sabadin et al. 2022). However, the efficiency of this strategy depends on the number of individuals included in the TS each cycle. Larger progenies (100–200) ensured more informative TS, whereas smaller progenies (25–50) produced limited TS that accelerated the decline in accuracy. This observation is consistent with findings from Werner et al. (2020), who showed that both size and composition of TS are decisive factors in sustaining GS performance.

The practical implication is that progeny size indirectly influences predictive ability through its effect on TS composition. While increasing marker density or refining models can improve GS accuracy to some extent (Endelman 2011; de Los Campos et al. 2013), our results suggest that ensuring sufficiently large and diverse progenies may be even more effective in preserving long-term accuracy. For breeders, this means that choices in progeny design have consequences not only for gain and variance but also for the stability of genomic predictions across cycles.

### Budget-constrained scenarios and trade-offs

When population size was capped at 4000 individuals per cycle, the optimal strategy shifted from large progenies to smaller ones combined with a greater number of crosses. In this context, progenies of 25 and 50 individuals delivered the highest cumulative gains, whereas progenies of 200 individuals—requiring only 20 crosses—showed much lower responses (Fig. 4a–f). This outcome underscores the importance of balancing depth (individuals per progeny) and breadth (number of crosses). With limited resources, increasing the number of crosses broadens the genetic base, maintains effective population size, and ensures continued access to novel allele combinations.

These findings align with previous simulation studies that evaluated allocation strategies in GS programs. Gorjanc et al. (2018) demonstrated that maintaining a large number of families helps reduce drift and sustain long-term responses, even when progeny size per cross is modest. Similarly, Muleta et al. (2019) highlighted that when resources are limited, distributing efforts across more crosses improves GS efficiency by maximizing the sampling of genetic variance. In empirical breeding programs, this principle has also been applied to optimize resource allocation, favoring breadth of sampling over depth when costs constrain population size (Bernardo 2020; Cobb et al. 2019).

Therefore, under budget constraints, smaller progenies offer a practical and efficient compromise. They reduce per-family sampling but increase the number of recombination events captured across the program, sustaining additive and epistatic variance over time. This highlights a crucial trade-off for breeding programs: while theoretical gains are maximized by large progenies, real-world resource limitations often necessitate prioritizing the number of crosses to achieve long-term sustainability.

### Response to selection and efficiency

Response to selection (RS) provided further insights into the efficiency of different progeny sizes across contexts. In theoretical scenarios, RS was highest with large progenies, reflecting their ability to exploit within-family variance and identify superior recombinants (Fig. 6). This agrees with classical expectations that larger progenies increase the probability of recovering transgressive segregants (Falconer and Mackay 1996; Bernardo 2020). By contrast, under budget-constrained conditions, RS peaked with smaller progenies (25–50 individuals), since the larger number of crosses increased between-family variance and expanded the sampling of allelic diversity (Fig. 7).

The trade-off observed between progeny size and RS highlights a central principle in breeding design: efficiency is context-dependent. In resource-unlimited scenarios, depth provides an advantage by increasing selection intensity within families, but under constraints, breadth ensures a more robust long-term trajectory. These echoes recent findings in wheat and maize GS simulations, where increasing the number of families improved genetic gain and stabilized prediction accuracy (Werner et al. 2020; Fritsche-Neto et al. 2024a).

Moreover, RS trends emphasize the importance of variance partitioning. In larger progenies, short-term RS is high but declines as within-family variance is rapidly exhausted. In smaller progenies under constrained resources, RS is lower per family but cumulative gain is higher because variance is distributed across a larger set of crosses. These dynamic highlights why optimal designs differ depending on whether the breeding objective is short-term acceleration or sustainable long-term progress. However, these conclusions are derived from simplified simulation settings, and their translation to operational pipelines should consider additional sources of biological and environmental complexity.

In this context, a limitation of this study is that we modeled epistasis using discrete levels and did not explicitly incorporate epistasis-by-environment interactions (E × Epi). In practical breeding programs, non-additive effects may vary across environments, which can influence the strength and, in some cases, the direction of the trade-offs reported here. Therefore, while our conclusions regarding depth–breadth allocation are robust within the modeled scenarios, extending these recommendations to multi-environment pipelines should explicitly account for E × Epi and related interaction components. Thus, future work should integrate multi-environment scenarios to assess how epistasis × environment interactions modify optimal progeny size and mating strategies for achieving genetic gains over time.

## Conclusion

In this study, we demonstrated that the optimum progeny size depends on the breeding context. Under theoretical scenarios without resource limitations, progenies of 200 individuals enabled greater exploitation of additive and non-additive genetic effects, resulting in superior gains across short-, medium-, and long-term horizons. This configuration was particularly effective in maximizing prediction accuracy and in converting both additive and epistatic variance into sustained genetic progress. From a practical standpoint, these results indicate that well-resourced programs can prioritize depth (larger progenies) to maximize long-term gain, especially when targeting traits influenced by epistasis.

In contrast, under budget-constrained conditions, the optimum progeny size was defined by the balance between the number of individuals per cross and the number of crosses performed. The most efficient strategies were 160 crosses with 25 individuals per progeny and 80 crosses with 50 individuals per progeny, which allowed broader exploration of genetic diversity without exceeding resource limitations. These arrangements provided more consistent medium- and long-term gains, highlighting that in resource-limited programs the most robust strategy is to prioritize the diversity of crosses rather than increasing progeny size. In practice, emphasizing breadth (more crosses with smaller progenies) also helps preserve genetic diversity, mitigate drift risk, and improve the stability of GS predictions.

Our results also reinforce the decisive role of epistasis in shaping selection trajectories. Moderate levels of epistasis favored the release of hidden additive variance, extending the response to selection and sustaining long-term gains even when initial prediction accuracy was lower. In contrast, high or antagonistic epistasis reduced or delayed progress, generating periods of stability. This demonstrates that the epistatic background of each population, combined with the selection strategy, is critical for long-term success.

Therefore, the optimum design of genomic selection programs should consider not only heritability and additive effects but also the magnitude of epistasis and the trade-off between progeny size and number of crosses. Such an integrated perspective is essential to maximize genetic gains efficiently and sustainably under different resource contexts.

## Data Availability

The datasets and scripts used for this study can be found in the supplementary material.
