# Correction: Optimizing progeny size and number of crosses under genomic selection: insights into additive and epistatic contributions to long‑term genetic gain

**DOI:** 10.1007/s00122-026-05187-9

**Published:** 2026-02-17

**Authors:** Jesimiel da Silva Viana, Júlio César DoVale, Roberto Fritsche-Neto

**Affiliations:** 1 Department of Crop Science, Federal University of Ceara, Fortaleza, Ceará Brazil; 2 North Carolina State University, Raleigh, NC USA

**Correction to: Theoretical and Applied Genetics (2026) 139:53** 10.1007/s00122-026-05164-2

In this article, the first author name was incorrectly published as “Jesimel da Silva Viana” instead of “Jesimiel da Silva Viana”.

The original article has been corrected.

